# Woody plant encroachment modifies carbonate bedrock: field evidence for enhanced weathering and permeability

**DOI:** 10.1038/s41598-023-42226-7

**Published:** 2023-09-18

**Authors:** Pedro A. M. Leite, Logan M. Schmidt, Daniella M. Rempe, Horia G. Olariu, John W. Walker, Kevin J. McInnes, Bradford P. Wilcox

**Affiliations:** 1 Department of Ecology and Conservation Biology, Texas A&M University, College Station, TX USA; 2 Department of Geological Sciences, Jackson School of Geosciences, University of Texas at Austin, Austin, TX USA; 3 Edwards Aquifer Authority, San Antonio, TX USA; 4 Texas A&M AgriLife Research and Extension Center, Texas A&M University, San Angelo, TX USA; 5 Department of Soil and Crop Sciences, Texas A&M University, College Station, TX USA

**Keywords:** Biogeochemistry, Environmental sciences, Hydrology, Geochemistry, Geomorphology, Hydrogeology, Ecosystem ecology

## Abstract

Little is known about the effects of woody plant encroachment—a recent but pervasive phenomenon—on the hydraulic properties of bedrock substrates. Recent work using stream solute concentrations paired with weathering models suggests that woody plant encroachment accelerates limestone weathering. In this field study, we evaluate this hypothesis by examining bedrock in the Edwards Plateau, an extensive karst landscape in Central Texas. We compared a site that has been heavily encroached by woody plants (mainly *Quercus fusiformis* and *Juniperus ashei*), with an adjacent site that has been maintained free of encroachment for the past eight decades. Both sites share the same bedrock, as confirmed by trenching, and originally had very few trees, which enabled us to evaluate how encroachment impacted the evolution of hydraulic properties over a period of no more than 80 years. Using in situ permeability tests in boreholes drilled into the weathered bedrock, we found that the mean saturated hydraulic conductivity of the bedrock was higher—by an order of magnitude—beneath woody plants than in the areas where woody plants have been continuously suppressed. Additionally, woody plant encroachment was associated with greater regolith thickness, greater plant rooting depths, significantly lower rock hardness, and a 24–44% increase in limestone matrix porosity. These findings are strong indicators that woody plant encroachment enhances bedrock weathering, thereby amplifying its permeability—a cycle of mutual reinforcement with the potential for substantial changes within a few decades. Given the importance of shallow bedrock for ecohydrological and biogeochemical processes, the broader impacts of woody plant encroachment on weathering rates and permeability warrant further investigation.

## Introduction

Woody plant encroachment (WPE) into grasslands and savannas, a land-cover change seen throughout the globe, has important implications for water and biogeochemical processes^1^. Its effects on the water budget include changes in not only evapotranspiration, but also infiltration rates^2, 3^. Enhanced soil infiltrability following WPE has been demonstrated in many locations^4–9^ and can result in dramatic changes to both streamflow and groundwater recharge^3, 10, 11^.

In some areas where soils are shallow, woody plants can utilize substantial amounts of rock moisture to sustain transpiration^12–15^. As plants tap into bedrock, enhanced water and CO_2_ fluxes along root channels may promote further rock dissolution, advancing weathering fronts and increasing bedrock permeability^16–19^. This process creates a positive feedback mechanism that, over time, improves the bedrock’s capacity to store moisture, making more water available to plants^20–23^.

It has long been recognized that trees are important agents of biochemical and biomechanical weathering, but the spatial and temporal scales at which such weathering processes take place have seldom been addressed^24, 25^. Similarly, very little is known about the extent to which WPE—a relatively modern phenomenon—accelerates weathering of bedrock. Findings from recent studies suggest that WPE increases bedrock weathering and permeability. For example, in Kansas, increases in stream solute concentrations in limestone catchments have been attributed to enhanced limestone weathering by WPE^26–29^, although this hypothesis was not directly confirmed. In our study, we explored this hypothesis at two adjacent sites in the Edwards Plateau of Texas—one site long maintained mostly free of WPE and the other heavily encroached.

## Study area

Our study took place between August 2020 and January 2021, at the Texas A&M Research Station near Sonora, TX, USA. The Station is located at approximately 700 m above sea level, in the southwestern portion of the Edwards Plateau—a major limestone formation that extends across much of West-Central Texas. Established for research purposes over 100 years ago, the 14-km^2^ Sonora Station has been subject to diverse land uses and management practices over the years. The Station also houses detailed historical records of these uses and practices, making it an ideal location for comparative research. The climate is semiarid, with a mean annual temperature of 18 °C and mean annual precipitation of around 560 mm, 70% occurring as rainfall between May and October^30^. Peak 15-min storm intensities of 90 mm/h have a return period of 2 years^31^.

The onset of WPE likely took place during the mid-1800s—in the wake of overgrazing and the suppression of natural fires—but it has accelerated in the last 60 years^10, 32^. It is difficult to know the exact vegetation composition before European settlement and the introduction of livestock, but historical accounts suggest that this region was mostly covered with perennial grasses; woody plants occurred more sparsely, and denser stands were mainly associated with rock outcrops, escarpments, and waterways^32^. Available records show that from the 1890s until the 1960s the region was heavily overgrazed, with stocking rates more than ten times the current rates^10^.

**Figure 1:**
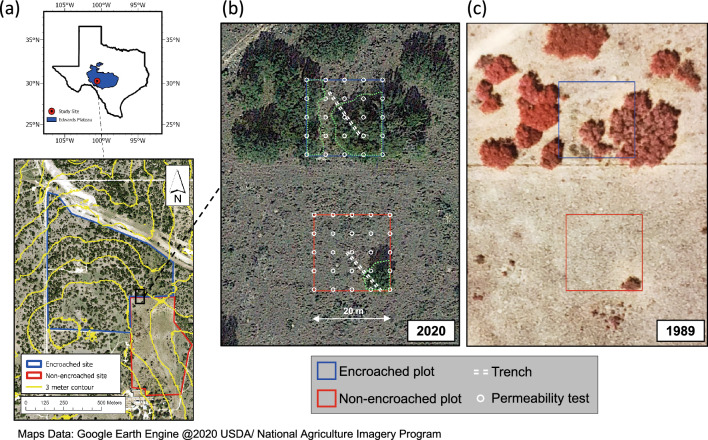
(**a**) Location of our study sites within the Edwards Plateau of Texas and the experimental plots (black outline). (**b**) and (**c**) The experimental plots and immediate vicinity in 2020 (Google Earth image) and in 1989 (aerial imagery). In (**b**), white circles indicate points where bedrock permeability tests were performed and dashed lines indicate the locations of our trenches. Within the plots, dotted green lines delimit the zones covered by woody plants.

The study area consists of a pair of sites: one site has undergone unrestrained encroachment by woody vegetation (encroached site), whereas the other has been continuously subjected to woody plant suppression since the 1940s (non-encroached site) (Fig. 1). Although the exact woody plant cover at the non-encroached site before the start of suppression is unknown, historical accounts for the Sonora Station suggest that very few trees were present. Until the 1970s, the suppression of woody plants at this site was carried out via sporadic mechanical removal, prescribed burns, and applications of herbicides, and since then by prescribed burns alone. In 1986, woody plant cover at the encroached and non-encroached sites was 7.6% and 5.4%, respectively, whereas by 2020 it was 19.3% and 4%, respectively (Figure S3). The dominant woody plant species at the encroached site are Ashe juniper (*Juniperus ashei*), Redberry juniper (*Juniperus pinchotii),* and Live oak (*Quercus fusiformis*). The few large woody plants at the non-encroached site consist mainly of scattered Live oak trees; a few small juniper saplings were also noted within the site. The intercanopy of both sites is largely dominated by a mixture of short and mid grasses and Prickly pear (*Opuntia spp.*). Both sites were grazed by sheep at high stocking rates from the late 1940s until the late 1960s. Since then, they have been grazed by sheep and goats at low stocking rates with periods of no grazing.

The topographies of the two sites are comparable and range from level ground to very gentle slopes (< 3%). Both sites have very shallow soils (< 50 cm) and are underlain by the Buda formation (a 15- to 30-m-thick layer of Upper Cretaceous, fine-grained, bioclastic, and chalky limestone). The water table lies at depths greater than 50 m^33^.

Within each site, we established a 20 m by 20 m experimental plot. These plots are 17 m distant from each other, are on near-level ground (no elevation differences between them), and are located on the same continuous bed. Both plots have a mixture of canopy and intercanopy cover (Fig. 1). The experimental plot within the encroached site (encroached plot) straddles two woody plant clusters separated by a narrow zone of intercanopy in the center and at the northern end. The plot within the non-encroached site (non-encroached plot) encloses mostly intercanopy cover with a single large Live oak tree in the southeast corner (Fig. 1). We determined that at the time of our study this oak tree was 54–57 years old, by counting the annual growth rings of a cross-section obtained from its main trunk (Figure S5). Woody plant cover on the plots and in their immediate vicinity was estimated (via the line intercept method) to be approximately 50% for the encroached plot and 4% for the non-encroached plot.

**Figure 2:**
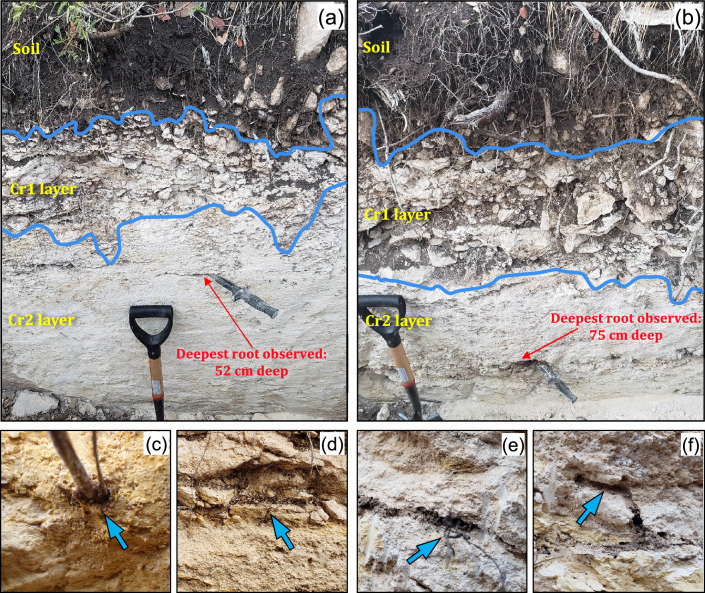
Profiles in the trench at the encroached site, at locations approximately 5 m apart and equally distant from the canopy dripline, in (**a**) an intercanopy zone and (**b**) a canopy zone. The knife is 20 cm long. (**c**) A 5-mm-thick root growing directly through the weathered limestone matrix. (**d**) Petrocalcic fragments between the Cr1 and Cr2 layers. (**e**) Wide fracture, filled with soil and woody roots, in the Cr2 layer. (**f**) Termite tunnels in the Cr2 layer.

At both sites, we measured the permeability of shallow limestone bedrock in the canopy and intercanopy locations (*Methods*; Fig. 1) and excavated trenches (*Methods*; Figs. 1 and 2) to document root-zone properties (such as depth to the weathered bedrock, maximum rooting depth, rock hardness, and matrix porosity).

## Results and discussion

### Bedrock permeability is much higher under woody plants

**Figure 3:**
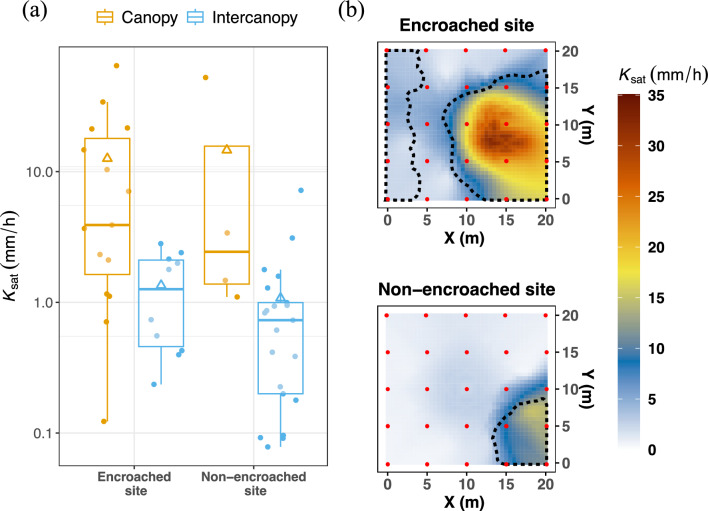
(**a**) Box plots showing the median, inter-quartile range, 95% confidence interval, and mean (triangles) of weathered bedrock *K*_sat_ in the canopy and intercanopy zones of the encroached and non-encroached sites. The y-axis was log10-scaled for improved visualization. (**b**) Interpolation maps of *K*_sat_ for weathered bedrock in the encroached and non-encroached sites. The dotted black lines delimit the areas covered by woody plants and the red dots indicate the points at which the permeability tests were performed (see also Fig. 1b).

Our results indicate that at both our study sites in the Edwards Plateau, WPE has significantly altered the hydraulic properties of the limestone bedrock. Two-way ANOVA results show that *cover* (canopy vs. intercanopy) was the only variable that had an effect on the mean saturated hydraulic conductivity (*K*_*sat*_; *p*-value = 0.001). Neither the *site* variable (non-encroached vs. encroached) nor the interaction term (cover*site) showed any effects (*p*-values > 0.7). Across both sites, the *K*_*sat*_ of the weathered bedrock was higher under woody plants (13 ± 18.6 mm/h) than in intercanopy zones (1.17 ± 1.4 mm/h). *K*_*sat*_ under woody plants ranged between 0.12 and 64.7 mm/h), while in the intercanopy it ranged between 0.08 and 7.2 mm/h (Fig. 3a and Table S1).

Maps of interpolated *K*_*sat*_ values indicate that for both sites, areas of high *K*_*sat*_ broadly overlapped with the aerial extent of canopy cover (Fig. 3b). For the encroached site, *K*_*sat*_ values were highest near the center of the largest woody plant cluster and decreased sharply with proximity to the canopy/intercanopy boundaries (canopy dripline). Importantly, the fact that in the non-encroached site *K*_*sat*_ was substantially higher under the oak tree (which was less than 60 years old) suggests that enhancement of bedrock permeability by encroaching woody plants can occur in a few decades.

A plausible argument could be made that woody plants have established preferentially in areas where *K*_sat_ was already high. Some support for this alternative hypothesis is offered by studies showing that survival rates improve when seedlings establish in areas with bedrock heterogeneities such as fractures and weathered joints^25, 34^. At the same time, other evidence suggests that woody plants are the main cause of the high *K*_sat_ values observed under their canopies. The fact that woody plant cover in the Edwards Plateau has been increasing continuously over the past few decades, reaching nearly 100% in some locations, by itself indicates that woody plant establishment is not necessarily predetermined by bedrock heterogeneities. Such heterogeneities are normally associated with topographic differences and bedding discontinuities^35, 36^, which were not factors in our study: both plots have the same elevation, and both are located on the same continuous bed. If pre-existing bedrock heterogeneities were the cause of high permeability, more points with high *K*_sat_ values would be expected in the intercanopy of the non-encroached plot (where woody plants would likely be present had they not been continuously suppressed). In addition, the fact that *K*_sat_ was substantially higher under the woody plants in the eastern portion of the encroached plot than under those in the western portion (Fig. 3b) offers support for the hypothesis that trees can improve weathered bedrock permeability: the trees in the eastern portion are larger and older than those in the western portion, as shown by both aerial imagery from 1989 and our field survey (Fig. 1c and Figure S4). This observation suggests that—like soil infiltration capacity^37, 38^—the effect of woody plants on limestone permeability can increase over time. Further investigations (e.g., including multiple sites representing chronosequences of WPE) could provide additional support to this hypothesis.

Other possible influences on bedrock permeability include the status (alive or dead), density, and species of woody plants. The eastern side of the encroached plot had several dead trees and higher tree density than the western portion (Figure S4). Greater density can mean more root biomass and higher competition for resources, which can drive trees to deepen their root profiles^39^. Over time, as woody plants die and their roots decay, preferential flow through root channels increases^40^. Finally, the relatively low *K*_*sat*_ on the western side of the encroached plot may be due not only to the smaller size and younger age of the trees but also to their species: Ashe junipers (Figure S4) are known to have shallower rooting depths than Live oaks^41^. To clarify the roles of all these possible influencing factors, additional research is needed.

### Mechanical and chemical weathering are enhanced under woody plants

While trees and shrubs have long been recognized as important agents of rock weathering and pedogenesis^24^, very few studies have provided strong evidence of bedrock weathering as a result of WPE, or that such weathering can occur over short timescales. Woody plants can accelerate both mechanical weathering, via root growth and expansion into fractures (wedging), and chemical weathering, by increasing the concentration of reactive substances such as organic and inorganic acids^42^. Carbonate rocks such as limestone are particularly susceptible to chemical weathering by acids; the concentration of these acids can be increased by plants through root respiration and the addition of organic matter^43^.

**Figure 4:**
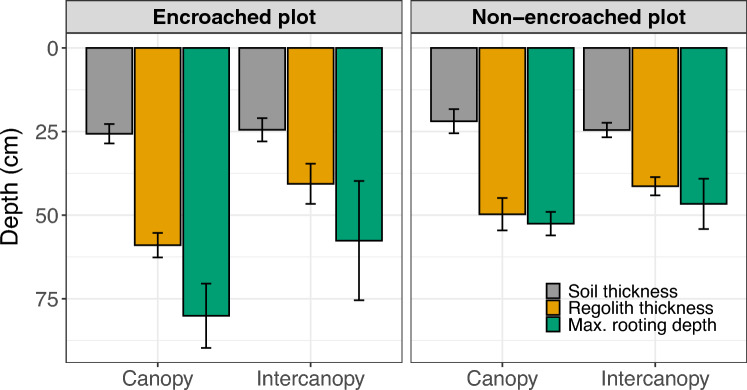
Bar graphs showing mean values of soil thickness (depth to the Cr1 layer), regolith thickness (depth to the Cr2 layer), and the maximum observed rooting depth by cover type (canopy vs. intercanopy) for the encroached and non-encroached sites. Error bars represent the 95% confidence interval.

In our study, we found evidence that shallow bedrock properties were influenced by woody vegetation (Fig. 4 and Table S2). For soil thickness (i.e., depth to the Cr1 layer), there were no significant differences between sites (p = 0.18) or between cover types (p = 0.91). However, for regolith thickness (i.e., depth to the Cr2 layer) while there were also no significant differences between sites (p = 0.24), there was a significant difference between cover types (*p* < 0.001): the mean regolith thickness in the canopy zones was 59 ± 7.8 cm for the encroached site and 49.7 ± 7.2 cm for the non-encroached site).

With respect to the maximum observed rooting depths, both the *site* and *cover* variables had significant effects (*p* < 0.001): the highest mean value was found for canopy cover in the encroached site (80.1 ± 20.6), and the lowest mean value was found for intercanopy cover in the non-encroached site (46.6 ± 11.2) (Fig. 4 and Table S2). No significant effects were found for interactions between the variables *site* and *cover*.

**Figure 5:**
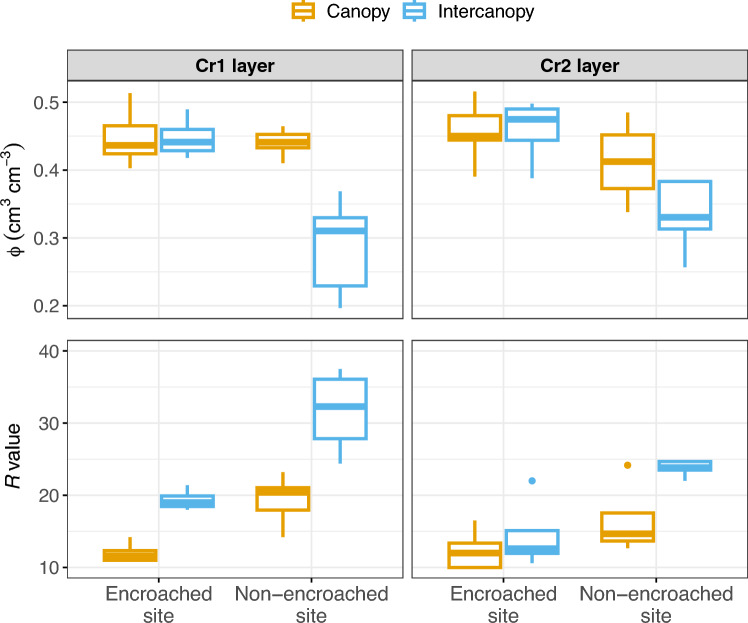
Box plots showing matrix porosity (Φ) and rebound values (*R*) of rock samples (obtained in situ with a Schmidt hammer) from the Cr1 and Cr2 layers of the encroached and non-encroached sites, by cover type (canopy vs. intercanopy).

Our analysis of limestone samples showed that matrix porosity ranged between 0.2 and 0.51 cm^3^cm^−3^ for the Cr1 layer and between 0.26 and 0.52 cm^3^cm^−3^ for the Cr2 layer. For the Cr1 layer, median porosity was 0.44 cm^3^cm^−3^ in both the canopy and intercanopy zones of the encroached site and in the canopy zone of the non-encroached site, but was significantly lower (0.31 cm^3^cm^−3^; *p* < 0.001) in the intercanopy zone of the non-encroached site (Fig. 5 and Table S2). For the Cr2 layer, median porosity values were highest in the encroached site, with no significant differences between canopy (0.45 cm^3^cm^−3^) and intercanopy (0.47 cm^3^cm^−3^) zones. For the non-encroached site, the median porosity of the canopy zone (0.41 cm^3^cm^−3^) was not statistically different (*p* = 0.11) from that of the intercanopy zones (0.33 cm^3^cm^−3^), but the intercanopy median porosity was significantly lower (p = 0.015) than that of the encroached site (Fig. 5).

For both sites, the median rebound (*R*) values (a proxy for rock hardness; these values are unitless and have a range of 10 to 100) for the Cr1 layer were significantly lower (*p* < 0.01) in canopy than in intercanopy zones (11.6 vs. 18.6 at the encroached site and 20.4 vs. 32.2 at the non-encroached site). Same-cover comparisons between the two sites showed significantly lower median *R* values for the encroached site (*p* < 0.01). Further, median *R* values for the Cr2 layer at the encroached site were similar for canopy and intercanopy zones (12 and 12.6) but were significantly different (*p* < 0.01) at the non-encroached site: 14.7 in the canopy zone versus 23.8 in the intercanopy zone. Rebound values and matrix porosity were inversely correlated (*p* < 0.001, R^2^ = 0.65; Figure S7).

Under the assumptions that (1) porosity across both sites was comparable before woody plant suppression started in the early 1940s, and (2) the documented differences in matrix porosity are primarily attributable to changes in vegetation, it can be inferred that WPE has led to limestone porosity gains of approximately 0.1 cm^3^cm^−3^ in less than a century. This finding contrasts starkly with the much slower rates of natural weathering of limestone, which would typically be on the order of several 1000 years for a comparable level of weathering to be reached^44^. Compared with grasslands, woodlands add more organic matter to the soil and have greater root respiration rates—two processes that increase concentrations of dissolved CO_2_ and organic acids in the vadose zone^27, 45^ and ultimately promote chemical weathering and thereby increase porosity.

The porosity gains documented in our study are substantially higher than those reported by Wen et al. (2021) for encroached and non-encroached sites in the Konza Prairie in Kansas, USA^26^. Using weathering models and stream solute concentrations, they estimated that it would take 300 years for WPE to result in a porosity gain of 0.01 cm^3^cm^−3^. It is important to note that weathering rates can vary with environmental conditions, geological settings, and bedrock characteristics. Our findings, while limited to two trenches, provide novel field evidence of substantial and relatively short-term bedrock weathering by encroaching woody plants. Additional investigations in other karst regions undergoing WPE are needed to determine whether our findings apply to other locations.

### Woody plants can enhance bedrock permeability through various mechanisms

Field evidence suggests that an important mechanism by which woody plants can enhance bedrock permeability is biomechanical root action, which creates preferential flow paths. Trench observations indicated that the canopy zones had a significantly thicker Cr1 layer and both the Cr1 layer and the petrocalcic film between it and the Cr2 layer were visually more broken down than in intercanopy zones (Figs. 2 and 4; also, see the *Trenching and pedologic observations* section in *Methods*). Additionally, the Cr2 layer in canopy zones displayed more abundant fractures and conduits, as well as root channels that were visually larger and extended to significantly greater depths. While roots mainly grow into existing rock fractures or highly weathered joints, ectomycorrhizal fungi associated with roots of many woody plants can penetrate narrow micropores of the rock matrix^46,47^. Some of these symbiotic microorganisms have been shown to promote biochemical weathering by excreting low-molecular-weight organic acids—earning them the name “rock-eating fungi”^48^. In other words, the high *K*_*sat*_ under canopies (especially where *K*_*sat*_ values were orders of magnitude higher than in intercanopy zones) is largely due to preferential flow through these features. Similar observations have been made by other researchers, who found that despite occupying less than 2% of the pore space, root channels contributed 93% of the saturated hydraulic conductivity of a weathered saprolite^19^. In addition to direct root action, woody plants might indirectly promote preferential flow by facilitating faunal activity. Biopores (e.g., termite tunnels) were visibly more abundant under trees (Fig. 2f). Invertebrate bioturbation can contribute to the high soil infiltration rates under woody plants^49, 50^, and this might also be the case for bedrock permeability.

Chemical weathering, by increasing matrix porosity, likely contributed to the enhancement of permeability rates. However, a paradox emerges in the case of the intercanopy zone of the encroached site: permeability remains comparatively low despite the fact that matrix porosity is as high as that found in the canopy zone (a feature we attribute to the dense woody plant cover surrounding the intercanopy) An indirect explanation for this reduced permeability could be the greater rock hardness in the intercanopy of both the encroached and non-encroached sites—as supported by our observation of generally higher *R* values in these zones (Fig. 5). Greater rock hardness means more resistance to root penetration and thereby lessened development of preferential flow paths via biomechanical weathering. Thus, while enhanced matrix porosity can contribute to increased permeability, if rock strength is high enough to restrict root-mediated biomechanical weathering, that factor might play a more pivotal role.

Another indirect way in which trees can promote chemical weathering and limestone permeability is by increasing soil infiltration capacity. When infiltration rates are higher and overland flow is lower, more water can reach the underlying limestone. At the Sonora Station, water has been shown to infiltrate five times faster and reach three times greater depths under junipers than in intercanopy soils^5^. As reactive water infiltrates deep through root channels and enlarged fissures, it accelerates carbonate dissolution and deepens the weathering front^20, 26^. Over time, more roots can penetrate the increasingly weathered rock, and the process becomes mutually reinforcing^24, 51^.

### The enhanced bedrock permeability brought about by woody plants has ecohydrological implications

In this study, we examined the extent to which WPE might have accelerated weathering of carbonate parent material in a semiarid climate. These observations, in combination with those from previous studies documenting changes in soil infiltrability following WPE^5, 52, 53^ provide strong evidence that WPE appreciably alters soils and substrates in karst terrain. These changes can have important implications for ecohydrological dynamics in these landscapes—in particular because they have the potential to modify (1) subsurface lateral flows, (2) deep percolation, and (3) groundwater recharge. Below we present support for these assertions.

#### Subsurface lateral flow

In semiarid regions, the main runoff process is generally considered to be infiltration-excess overland flow, which is controlled by the infiltrability of the topsoil^54^. However, where soils are shallow, bedrock permeability can also exert a strong control on runoff. If permeability is low, perched water tables can form, leading to saturation-excess overland flow and shallow subsurface lateral flow^52, 55^. Even when soils are unsaturated, high-intensity storms can generate subsurface flow, as rapid preferential flow through soils encounters low-permeability bedrock^52, 56^.

High-intensity, hillslope-scale rainfall simulation experiments in the Edwards Plateau have demonstrated that shallow subsurface flow can be an important component of runoff generation in these landscapes, particularly under tree canopies^52, 53^. These simulations produced no surface runoff from canopy zones but did produce large amounts of fast lateral subsurface flow through bedrock fractures filled with woody roots. From intercanopy zones, in contrast, the simulations produced substantial surface runoff and little subsurface flow (most of the latter through the interface between soil and bedrock)^52, 53^. Our findings of higher rates of weathering and limestone permeability beneath woody plants provide a mechanistic explanation for the results of those simulation experiments, showing that trees can create preferential flow networks in weathered bedrock and thereby promote shallow subsurface lateral flows.

#### Deep percolation

The acceleration of weathering and promotion of preferential flows brought about by WPE will likely contribute to deep percolation within the root zone. For example, continuous measurements of rock moisture at our study site showed that following a 95-mm storm event, the water content of fractures in the Cr2 layer (at 60 cm and 80 cm deep) peaked within 2–3 h of peak rainfall. At the same depths, the water content of the unfractured limestone matrix would take days or even weeks to show changes^57^. Further evidence that woody plants can promote deep percolation comes from observations of trees channeling large amounts of water deep into the bedrock vadose zone via a combination of stemflow and preferential flow along fractures and root channels^58, 59^.

By enhancing the recharge of deeper water pools within the root zone, trees might increase their chances for long-term survival. For example, work in the Edwards Plateau has shown that juniper and oak trees on shallow soils underlain by fractured bedrock have lower mortality rates than those on deep soils^60^—evidence not only of their ability to use rock moisture, but that it is a valuable resource for maintaining basic physiological functions during dry periods^14, 22, 61^. We postulate that these plants’ ability to also alter bedrock within their lifetime will favor both their survival rates and overall fitness—a clear example of niche construction^62^. When bedrock is permeable and porous, which facilitates deep water percolation and high available water storage, woody plants’ competition with shallower-rooted grasses might be reduced. Young seedlings are more affected by this competition^63^ and could reap the most benefit, as their survival rates improve when they have access to bedrock heterogeneities such as fissures and joints^25, 34^. Contrary to soil modifications, which may have relatively short-lived geomorphological signatures, rock weathering is irreversible and persists long after woody plants have died or been removed—an illustration of ecosystem engineering ^24, 64^.

#### Groundwater recharge

Where bedrock permeability is high, vertical flows can be intensified, facilitating deep drainage and opportunities for direct groundwater recharge. Multiple factors govern these processes, including climate, geological setting, rooting depths, and depth to the water table^2, 65^. In semiarid karst settings, recharge occurs mainly following high-intensity storms, when water bypasses much of the vadose zone via preferential flow^66^. However, if most of the infiltrated water is stored within the root zone and transpired by vegetation, direct recharge will be minimal^65, 67^.

While direct recharge might be negligible in many semiarid regions, focused recharge in topographic depressions and karst features can be substantial^55, 68^. In this context, the effects of higher bedrock permeability and porosity brought about by WPE can be complex and hard to predict. Increased bedrock permeability could help explain the high levels of subsurface lateral flow observed under woody plants^52^. Such flows can bypass much of the soil and rock matrix^57, 58^ and drain into ephemeral springs and streams, where recharge occurs via transmission losses^68^. Alternatively, higher bedrock permeability and porosity could result in more water being stored on-site and being transpired instead of becoming runoff, consequently lowering focused recharge.

## Conclusion

Our study provides evidence of the profound impact of WPE on the hydraulic properties of carbonate bedrock. Through biomechanical root action and biochemical processes, woody plants create preferential flow networks in weathered bedrock. This altered subsurface hydrology has important implications for ecohydrological dynamics, potentially affecting subsurface lateral flows, deep percolation, and groundwater recharge. As WPE continues to shape landscapes, understanding its effects on bedrock permeability and water pathways becomes increasingly important for managing water resources in semiarid ecosystems, particularly those with shallow soils. Further research is warranted to explore the interplay between WPE, bedrock weathering, and water dynamics across diverse climatic and geological settings.

## Methods

### Trenching and pedologic observations

To investigate the nature of the limestone bedrock, we excavated two trenches, one in each site (by means of a backhoe with a 90-cm-wide excavator bucket). We selected the trenching method because it is cost-effective and allowed us to not only take samples but also collect in situ data on rock hardness, depth to different horizons, and rooting depths. The trenches also enabled us to confirm that both plots were on the same continuous bed. In the encroached site, the trench was 16 m long, 11 m of which traversed a canopy zone and 5 m traversed an intercanopy zone. In the non-encroached site, the trench was 15 m long–5 m traversing the canopy of the Live oak tree and 10 m traversing intercanopy (Fig. 1). Whereas the encroached site trench was relatively easy to excavate to a depth of 1.5 m, the limestone beneath the non-encroached site was considerably harder to excavate, particularly in the intercanopy zone. The maximum depth attained in the non-encroached-site trench was 1 m, and only a 10-m-long section was deeper than 50 cm. That section was used for our rock sampling and analysis (described below).

In both trenches, three major layers could be easily distinguished: soil, a highly fractured and broken limestone layer (Cr1 layer), and a much less fractured limestone layer (Cr2 layer) (Fig. 2). Soils are very shallow Calciustolls, consisting of an A horizon (5–15 cm thick) of dark brown, silty clay loam and a Bw horizon (5–15 cm thick) of brown clay containing more than 50% rounded limestone cobbles and stones. Along with woody plant roots, most of the non-woody roots (from grasses, forbs, and cacti) were found in the A horizon. Woody roots were also abundant in the Bw horizon and in the weathered limestone. The Cr1 layer is 20–40 cm thick and consists of highly fractured and moderately hard limestone. In canopy zones, this layer was visibly more broken down, had wider fractures and conduits (often infilled with soil), and had thicker and more plentiful woody roots (Fig. 2b) than in intercanopy zones (Fig. 2a). The Cr2 layer consists of moderately soft to hard limestone with far fewer fractures and woody roots than the Cr1 layer. In canopy zones, the Cr2 layer was relatively soft and friable (fragments easily broken by hand), with medium-size woody roots growing both in fractures and directly through the matrix (Fig. 2c); in intercanopy zones, the Cr2 layer was harder (not easily broken by hand), with fewer and thinner woody roots, mostly growing in narrow fractures. This layer also exhibited occasional widened fractures and conduits infilled with soil and containing many woody roots, more commonly in canopy zones (Fig. 2e). Faunal biopores (e.g., termite tunnels) were observed in both weathered limestone layers but were clearly more abundant underneath trees (Fig. 2f). The boundary between the two layers was identified as a thin film of very hard limestone believed to have a pedogenic (petrocalcic) origin—formed by the dissolution and transport of carbonates from the soil and Cr1 layers and subsequent reprecipitation onto the less permeable Cr2 layer^69^. This thin film was generally broken down, especially in canopy zones (Fig. 2d).

### Limestone permeability

In August 2020, we drilled a total of 25 boreholes within each plot, using a compressed-air rock drill (Ingersoll‐Rand, Piscataway, NJ, USA) with a 7.62-cm-diameter bit. The boreholes were drilled to the full reach of the drill (61 cm), at the nodes of a grid with 5-m spacing (Fig. 1), and each succeeded in attaining weathered bedrock. The boreholes were then cleaned—first by manual removal of large debris, then with a planer auger to remove finer debris and level the bottom, and finally with a leaf blower having an extended nozzle to remove dust and very fine debris. Boreholes located under woody plants were classified as “canopy,” and those located in open spaces (i.e., no tree cover) as “intercanopy”.

We used saturated hydraulic conductivity (*K*_*sat*_) as our metric for weathered bedrock permeability. To ensure continuous and automatic measurement in the field, we used constant-head well permeameters (Supplementary materials; Figure S1). These permeameters are widely employed for measuring the permeability of soils and weathered parent material^70, 71^. Because water ponds inside the well, the infiltration process tends to be controlled by preferential flow through empty root channels, conduits, and fractures^18^. Thus, *K*_*sat*_ data obtained via well permeameter methods are particularly useful for evaluating changes in macrostructure^72^, such as mechanical weathering. We opted to use a relatively small (5-cm) head to ensure that measurements were performed within the weathered bedrock, which was confirmed by visual inspections. However, from the boreholes it was difficult to distinguish between the Cr1 and Cr2 layers, and some tests likely included a combination of the two, which might have contributed to some of the large variability in *K*_sat_.

During operation, changes in gas pressure in the permeameter were monitored by a datalogger and recorded every 30 s (Figure S2). The change in the permeameter’s water level and gas pressure are related as follows**:**

1 $$\Delta {P}_{g}= -pg\Delta h$$

where Δ*P*_*g*_ is the change in gas pressure in the permeameter, *p* is the density of water, *g* is the acceleration of gravity, and Δ*h* is the water head drop in the permeameter. Thus, the steady-state flow rate (*Q*) can be obtained by

2 $$Q=\Delta {P}_{g} ({r}^{2})\pi /pg\Delta t)$$

where r is the radius of the reservoir and Δ*t* is the change in time (Figure S2).

Then, *K*_*sat*_ can be calculated as

3 $$K_{sat} = CQ/\left( {2\pi H^{2} + \pi a^{2} C + \frac{2\pi H}{{\alpha^{*} }}} \right)$$

where *H* is the ponded water head in the well, *a* is the well radius, α^*^ is a capillary length factor dependent on texture–structure category, and *C* is the shape coefficient, calculated as

4 $$C = \left[ {\left( \frac{H}{a} \right)/\left( {Z_{1} + Z_{2} \left( \frac{H}{a} \right)} \right)} \right]^{{Z_{3} }}$$

where *Z*_*1*_, *Z*_*2*,_ and *Z*_*3*_ are empirically determined constants that depend on the α^*^ value. Since the precise value of α* was not known, we used an estimated value of 0.01 cm^−1^, which is suitable for mediums with very strong capillarity^71^. Although this approximation may introduce uncertainty in the absolute *K*_*sat*_ values, the relative differences in *K*_*sat*_ remain unaffected and valid regardless of the selected α* value.

Measurements were terminated after the permeameter was entirely evacuated, or—in cases when infiltration was particularly slow—after at least 2 h of monitoring. While early termination of tests could have resulted in *K*_sat_ overestimation, the infiltration curves (e.g., Figure S2) indicated that steady-state was always achieved.

### Other properties of limestone

In each of the two trenches, at 50-cm intervals, we recorded the depth to the Cr1 layer (soil thickness), the depth to the Cr2 layer (regolith thickness), and the deepest woody root observed (Figure S6). To estimate the hardness of the limestone surface, we performed in situ measurements of rebound values (*R*) using an H-2975 Schmidt hammer device (Humboldt Scientific, Raleigh, NC). This device measures the rebound of a spring-loaded metal piston hitting a surface at a defined energy. It has been used extensively in geomorphological studies, which have shown that the *R* values directly reflect the surface hardness of the rock and are highly correlated with rock compressive strength and degree of weathering^73^. Before performing a test, we smoothed the rock surface with a medium-grained grinding stone. Then, at each point selected for later collection of a rock sample, we carried out the tests—at a right angle to the flat surface and avoiding visible fractures. We took five to six measurements at each point and averaged the resulting *R* values.

Next, we took cobble-size samples of the Cr1 and Cr2 layers at 1-m intervals. Samples from the Cr1 layer were taken from the middle of the layer, while samples from the Cr2 layer were taken at a depth of 20 cm below the top of the layer (Figure S6). In the laboratory, a piece of thread seal tape was tied around the samples, which were placed in a water container and left there for 48 h to achieve saturation. Then, while hanging from the tape, the samples were submerged one by one in a water-filled container mounted on top of a precision scale (0.01-g resolution), and the weight change was recorded. Because the change in weight is equivalent to the volume of water displaced by the sample (i.e., water density ≅ 1 g cm^−3^), the sample’s volume (V [cm^3^]) can be readily obtained with an accuracy of 0.01%—the same level of accuracy as that of pycnometers^74^. Finally, we calculated dry bulk density (ρ_b_ [g cm^−3^]) and matrix porosity (Ф [cm^3^ cm^−3^]), respectively, as follows:

5 $${\rho }_{b}=\frac{{m}_{dry }}{V}$$

6 $$\Phi = \left( {1 - \frac{{\rho_{b} }}{{\rho_{s} }}} \right)$$

Where m_dry_ is the dry mass (obtained by oven drying the sample at 105 °C for 48 h) and ρ_s_ is the particle density of limestone, for which the value of 2.66 g cm^−3^ was adopted^75^.

### Statistical analyses

To test the null hypothesis of no significant differences among the groups and no interaction effect, we performed a two-way ANOVA to evaluate the significance of (1) effects of the predictor variables *cover* (canopy vs. intercanopy) and *site* (encroached vs. non-encroached), and (2) the effect of their interaction on the response variable. This procedure was adopted for data conforming to parametric assumptions (log10-transformed *K*_*sat*_, depth to Cr1, depth to Cr2, and maximum rooting depth). Even after various transformations, the porosity and *R* values did not conform to assumptions of normality and equal variance**,** and for this reason we used the non-parametric Mann–Whitney test to identify significant differences between median values. All analyses were performed in R version 4.1.0. with a significance level of 0.05. To visualize the spatial trends of *K*_*sat*_ at the plot scale, we created 2-d interpolation maps using the loess() function of the R package ‘stats’.

### Supplementary Information

Supplementary Information 1.

Supplementary Information 2.

Supplementary Information 3.

Supplementary Information 4.

## Data Availability

The datasets generated and/or analyzed during the current study are available as Supplementary information files.
