# Supplementary material for: Woody plant encroachment modifies carbonate bedrock: field evidence for enhanced weathering and permeability
Source: Sci Rep. 2023 Sep 18;13:15431. doi: 10.1038/s41598-023-42226-7 (PMC10507015; doi:10.1038/s41598-023-42226-7)
Supplement: Supplementary file 4 — Supplementary Information 4. [file 41598_2023_42226_MOESM4_ESM.docx]

## Woody plant encroachment modifies carbonate bedrock: field evidence for enhanced weathering and permeability

Pedro A. M. Leite,*^1^ Logan M. Schmidt,^2,3^ Daniella M. Rempe,^2^ Horia G. Olariu, ^1^ John W. Walker,^4^ Kevin J. McInnes,^5^ Bradford P. Wilcox^1^

**^1^**Department of Ecology and Conservation Biology, Texas A&M University, College Station, Texas, USA. **^2^**Department of Geological Sciences, Jackson School of Geosciences, University of Texas at Austin, Austin, Texas, USA. **^3^** Edwards Aquifer Authority, San Antonio, Texas, USA. **^4^**Texas A&M AgriLife Research and Extension Center, Texas A&M University, San Angelo, Texas, USA. **^5^**Department of Soil and Crop Sciences, Texas A&M University, College Station, Texas, USA.

*Corresponding author (email: pedroleite@tamu.edu)

**Supplementary materials**

*Constant head well permeameter*

Our permeameter design was based on the devices described by Constantz and Murphy (1987) and Leite et al. (under review). The main reservoir consists of a 2-inch (5.08 cm)-diameter, 1-m-long PVC pipe sealed at its upper end by a rubber cap. A steel clamp over the cap and application of vacuum grease ensures an airtight seal. The cap is equipped with a small screw eye hook that is attached with a piece of wire to a HOBO U20L pressure transducer datalogger (Onset, Bourne, MA, USA). The lower end of the pipe is attached (via a 2-inch-to-1-inch reducer coupling) to a 1-inch (2.54 cm)-diameter, 10-cm-long schedule 40 PVC pipe, which is in turn attached to a PVC ball valve and a 1-inch-diameter, 70-cm-long PVC pipe. The lower end of this pipe is fitted with a perforated cap that serves as the outflow port. All PVC parts are glued together with PVC cement. The reservoir assembly is mounted on an adjustable tripod and secured with two steel clamps (Figure S1).


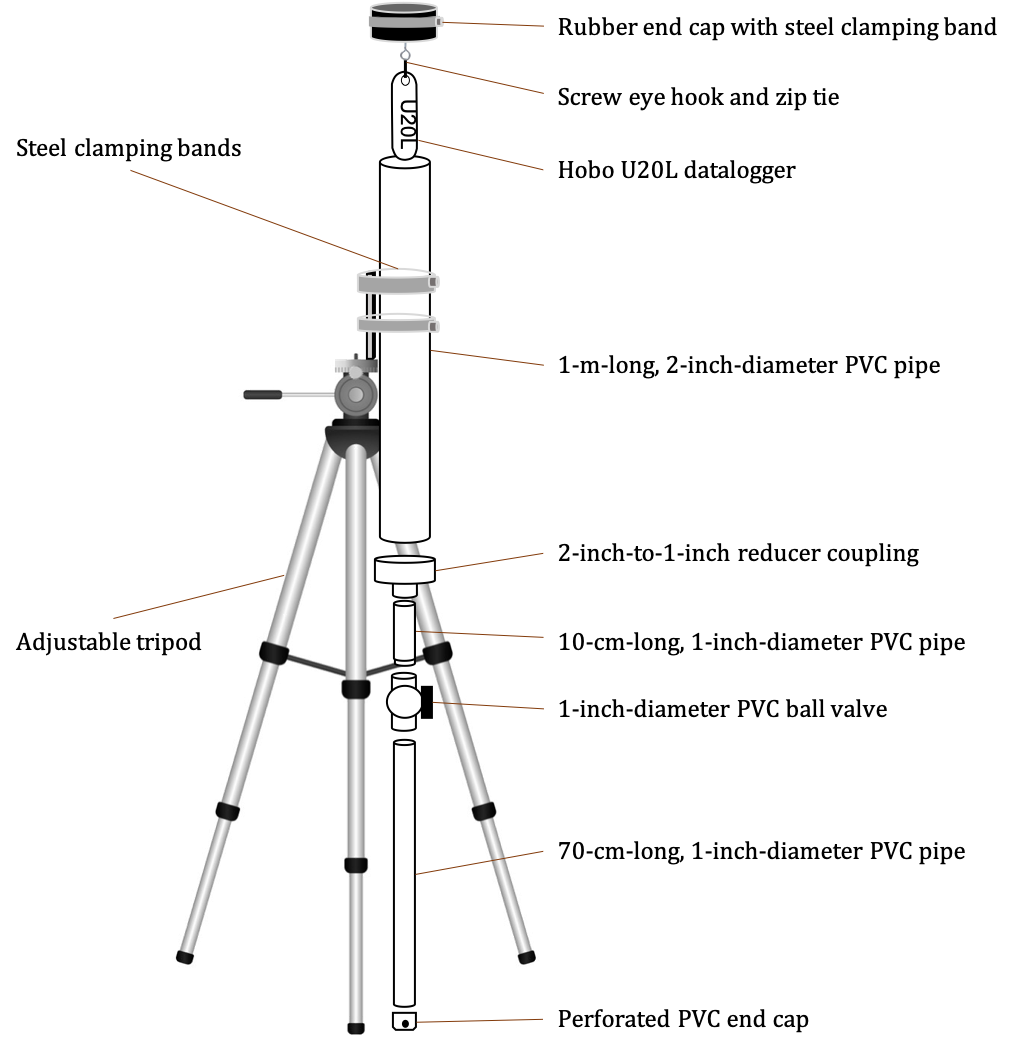


Figure S1: Schematic of the permeameter assembly.

Figure S2: Changes in gas pressure (*P*g) over time (t) recorded by the datalogger during a permeability test. (A): atmospheric pressure before the start of the test; (B): pressure when the valve is opened and the test begins, creating a sudden pressure drop that is proportional to the head of water in the permeameter; (C): gradual increase in pressure caused by air bubbles that enter the permeameter as water infiltrates into the well (initial infiltration rates are higher and pressure increases faster); (D): constant pressure, attained after some time (a few minutes in this case), indicating steady-state infiltration; (E): abrupt change in slope (pressure starts to increase faster) because water has passed from the 2-inch-diameter pipe into the 1-inch-diameter pipe; (F): atmospheric pressures are recorded as in (A), the permeameter now being completely evacuated. Data from either D or E can be used to calculate steady-state flow rates ($Q$). If calculated from D, $Q$ would be based on a reservoir radius (r) of 1 inch (2.54 cm) (see Equation (2) in the *Methods* section); if calculated from E, $Q$ would be based on an r of 0.5 inch (1.27 cm).

*Vegetation surveys*

Figure S3: Woody plant canopy cover at our sites in 1986 and 2020. The data was acquired from the Rangeland Analysis Platform (RAP) (<https://rangelands.app>). The black rectangle indicates the location of our plots.

Figure S4: Locations of woody plants within and up to 5 m beyond the boundaries (dashed lines) of the plots on the encroached and non-encroached sites. The Y axes are N–S oriented.

Figure S5: Cross section used for estimating the age of the oak tree at the non-encroached site. The total number of dark rings counted ranged between 54 and 57 (1 ring = 1 year). Uncertainty arises from difficulty in identifying some of the rings, especially in the sapwood area. Blue arrows indicate 10-year intervals.

*Trenching and pedologic observations*

**

Figure S6: depths (y axis) and relative distances along trenches (x axis) where depth measurements were performed and bedrock samples were taken for porosity analysis. The black vertical line indicates the location of the canopy dripline.

Figure S7: Correlations between *in-sit*u rebound values (*R*) and matrix porosity (ϕ) of limestone samples obtained from the Cr1 and Cr2 layers.

Table S1: Descriptive statistics of *K_sat_* (mm h^-1^) ­data for weathered bedrock in the encroached and non-encroached sites.

| **Site** | **Cover** | **N** | **Mean** | **Median** | **SD** | **Max** | **Min** |
| --- | --- | --- | --- | --- | --- | --- | --- |
| Encroached | canopy | 15 | 12.6 | 3.91 | 17.54 | 64.7 | 0.12 |
|  | intercanopy | 10 | 1.35 | 1.26 | 0.97 | 2.81 | 0.24 |
| Non-Encroached | canopy | 4 | 14.62 | 2.44 | 25.29 | 52.52 | 1.1 |
|  | intercanopy | 21 | 1.08 | 0.73 | 1.58 | 7.2 | 0.08 |

Table S2: Descriptive statistics of the depths (cm) to the Cr1 and Cr2 layers and the maximum observed rooting depth by cover type (canopy vs intercanopy) for the encroached (E) and non-encroached (NE) sites.

| **Property** | **Site** | **Cover** | **N** | **Mean** | **Median** | **SD** | **Max.** | **Min.** |
| --- | --- | --- | --- | --- | --- | --- | --- | --- |
| Depth to Cr1 | E | canopy | 20 | 25.7 | 24 | 6.2 | 38 | 15 |
|  |  | intercanopy | 11 | 24.5 | 23 | 5.2 | 35 | 18 |
|  | NE | canopy | 11 | 21.9 | 22 | 5.4 | 30 | 13 |
|  |  | intercanopy | 11 | 24.5 | 26 | 3.2 | 28 | 18 |
| Depth to Cr2 | E | canopy | 20 | 59.0 | 57.5 | 7.8 | 79 | 48 |
|  |  | intercanopy | 11 | 40.6 | 40 | 8.9 | 55 | 28 |
|  | NE | canopy | 11 | 49.7 | 51 | 7.2 | 58 | 31 |
|  |  | intercanopy | 11 | 41.4 | 40 | 4.1 | 51 | 38 |
| Maximum observed root depth | E | canopy | 20 | 80.1 | 73 | 20.6 | 118 | 52 |
|  |  | intercanopy | 11 | 57.6 | 52 | 26.5 | 109 | 33 |
|  | NE | canopy | 11 | 52.5 | 53 | 5.2 | 65 | 47 |
|  |  | intercanopy | 11 | 46.6 | 41 | 11.2 | 69 | 37 |

Table S3: Descriptive statistics of matrix porosity (Φ [cm^3^ cm^-3^]) and rebound values (R) of rock samples from the Cr1 and Cr2 layers of the encroached (E) and non-encroached (NE) sites, by cover type (canopy vs intercanopy).

| **Property** | **Layer** | **Site** | **Cover** | **N** | **Mean** | **Median** | **SD** | **Max.** | **Min.** |
| --- | --- | --- | --- | --- | --- | --- | --- | --- | --- |
| Φ | Cr1 | E | canopy | 9 | 0.452 | 0.436 | 0.040 | 0.513 | 0.403 |
|  |  |  | intercanopy | 4 | 0.447 | 0.441 | 0.031 | 0.489 | 0.418 |
|  |  | NE | canopy | 6 | 0.440 | 0.441 | 0.019 | 0.465 | 0.410 |
|  |  |  | intercanopy | 5 | 0.287 | 0.310 | 0.072 | 0.369 | 0.197 |
|  | Cr2 | E | canopy | 11 | 0.459 | 0.450 | 0.036 | 0.516 | 0.390 |
|  |  |  | intercanopy | 4 | 0.459 | 0.475 | 0.049 | 0.498 | 0.388 |
|  |  | NE | canopy | 4 | 0.412 | 0.412 | 0.064 | 0.485 | 0.338 |
|  |  |  | intercanopy | 5 | 0.334 | 0.330 | 0.054 | 0.385 | 0.257 |
| R value | Cr1 | E | canopy | 9 | 11.94 | 11.60 | 1.14 | 14.20 | 10.80 |
|  |  |  | intercanopy | 5 | 18.68 | 18.60 | 1.97 | 21.40 | 16.00 |
|  |  | NE | canopy | 6 | 19.43 | 20.40 | 3.21 | 23.20 | 14.20 |
|  |  |  | intercanopy | 4 | 31.63 | 32.30 | 6.04 | 37.50 | 24.40 |
|  | Cr2 | E | canopy | 11 | 12.10 | 12.00 | 2.23 | 16.50 | 10.00 |
|  |  |  | intercanopy | 4 | 14.45 | 12.60 | 5.12 | 22.00 | 10.60 |
|  |  | NE | canopy | 4 | 16.54 | 14.67 | 5.20 | 24.17 | 12.67 |
|  |  |  | intercanopy | 5 | 23.77 | 23.83 | 1.13 | 24.83 | 22.00 |

**References**

1. Constantz, J. & Murphy, F. An Automated Technique for Flow Measurements from Mariotte Reservoirs. *Soil Science Society of America Journal* **51**, 252–254 (1987).

2. Leite, P. A. M., DiPrima, S., Schmidt, L. M., Wilcox, B. P. A simple constant-head infiltrometer automated with a user-friendly pressure datalogger (under review).
